# Supplementary material for: CRISPR/Cas9-Mediated Targeted Mutagenesis of BnaCOL9 Advances the Flowering Time of Brassica napus L
Source: Int J Mol Sci. 2022 Nov 29;23(23):14944. doi: 10.3390/ijms232314944 (PMC9740695; doi:10.3390/ijms232314944)
Supplement: Supplementary file 1 [file ijms-23-14944-s001.zip › suplement figure.pdf]

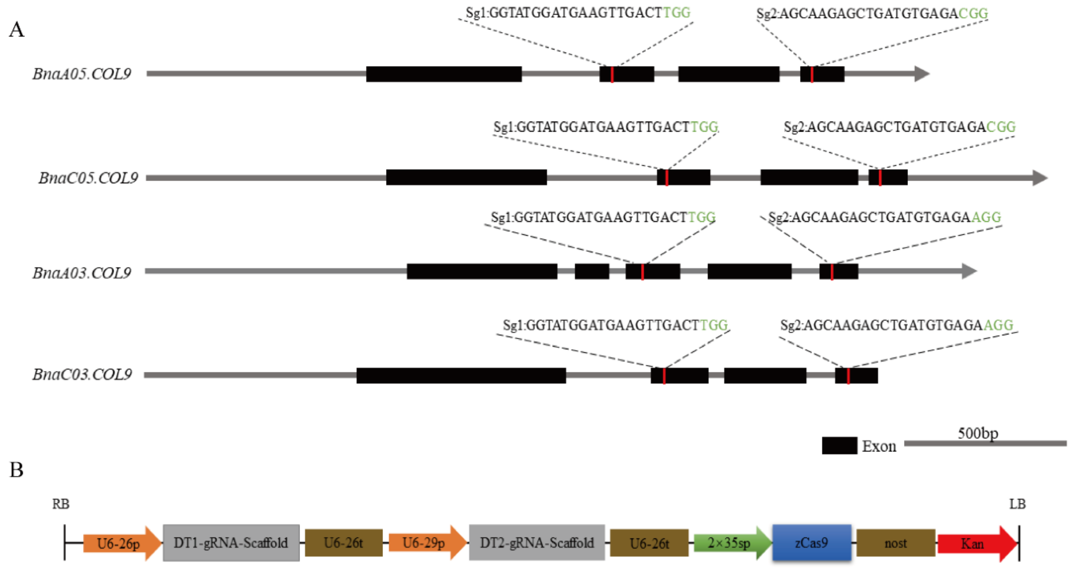

Figure S1 The target site in the *BnaCOL9* gene and the structure of the CRISPR/Cas9 vector. (A) Exon–intron structure of *BnaAO5COL9*, *BnaCO5COL9*, *BnaAO3COL9,* and *BnaC03COL9*. Black boxes indicate exons and red stripes are editing sites. Continuous lines indicate introns and untranslated regions. Nucleotide sequences indicate regions targeted by the sgRNAs designed in this study; nucleotides in green indicate the proto-spacer adjacent motif. (B) Schematic of the T-DNA region of the targeting vector designed for mutagenesis of the *BnaCOL9* genes using the CRISPR/Cas9 system. U6-26p and U6-29p indicate the Arabidopsis U6 promoter. U6-26t indicates the Arabidopsis U6 terminator. DT1-gRNA-Scaffold and DT2-gRNA-indicate sg1 and sg2. Cas9 was expressed by the 35S promoter. Red arrows indicate the kanamycin resistance gene. LB, left border; RB, right border.

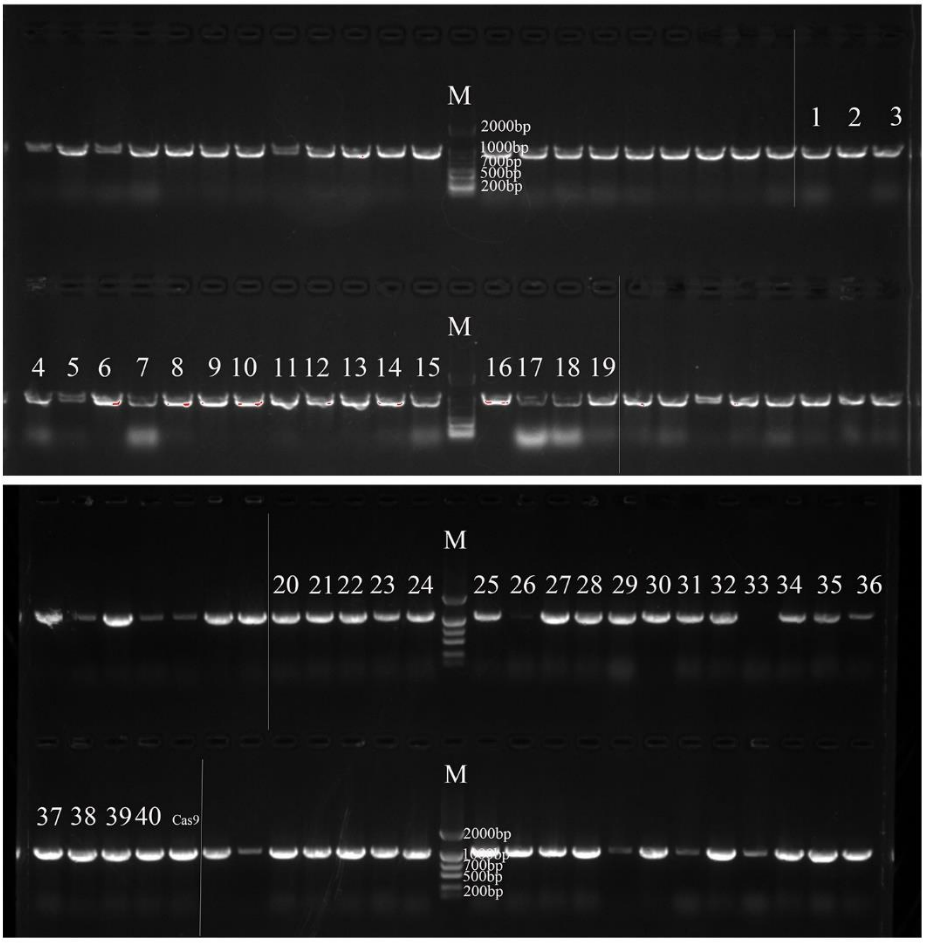

Figure S2 Positive identification of T0 regenerated plants via PCR using Cas9F/R primers. Cas9 was used as a positive control. 1-40 represent regenerated transgenic plants, respectively.

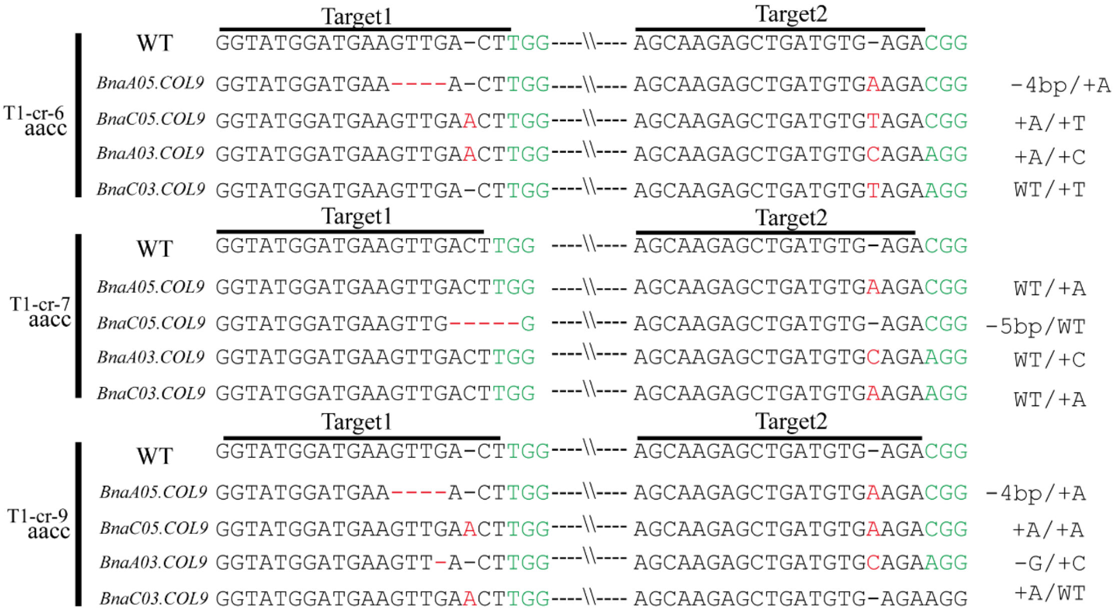

Figure S3 T1-cr-6, T1-cr-7, and T1-cr-9 T1 generation editing type. sequence analysis of target sites in knockout lines of three mutants with wild-type (WT) sequences shown at the top (the target site is below the black line the green font represents the PAM sequence, red fonts indicate insertions, and red short dashed lines indicate deletions.
